# Generalist genes and cognitive abilities in Chinese twins

**DOI:** 10.1111/desc.12022

**Published:** 2013-02-07

**Authors:** Bonnie Wing-Yin Chow, Connie Suk-Han Ho, Simpson Wai-Lap Wong, Mary MY Waye, Dorothy VM Bishop

**Affiliations:** 1 Department of Applied Social Studies, City University of Hong KongHong Kong; 2 Department of Psychology, The University of Hong KongHong Kong; 3 Department of Psychological Studies, The Hong Kong Institute of EducationHong Kong; 4 School of Biomedical Sciences, The Chinese University of Hong KongHong Kong; 5 Department of Experimental Psychology, University of OxfordUK

## Abstract

This study considered how far nonverbal cognitive, language and reading abilities are affected by common genetic influences in a sample of 312 typically developing Chinese twin pairs aged from 3 to 11 years. Children were individually given tasks of Chinese word reading, receptive vocabulary, phonological memory, tone awareness, syllable and rhyme awareness, rapid automatized naming, morphological awareness and orthographic skills, and Raven's Colored Progressive Matrices. Factor analyses on the verbal tasks adjusted for age indicated two factors: Language as the first factor and Reading as the second factor. Univariate genetic analyses indicated that genetic influences were substantial for nonverbal cognitive ability and moderate for language and reading. Multivariate genetic analyses showed that nonverbal cognitive ability, language and reading were influenced by shared genetic origins, although there were specific genetic influences on verbal skills that were distinct from those on nonverbal cognitive ability. This study extends the Generalist Genes Hypothesis to Chinese language and reading skills, suggesting that the general effects of genes could be universal across languages.

## Introduction

Individual variations and change in general cognitive, language and reading abilities tend to inter-correlate, suggesting possible common etiology in their development (Rhemtulla & Tucker-Drob, 2011). Substantial genetic overlap between these cognitive abilities has been demonstrated, supporting the Generalist Genes Hypothesis, which predicts that the same set of genes largely influences diverse domains of cognitive abilities (Plomin & Kovas, 2005). To date, the Generalist Genes Hypothesis has been tested on alphabetic languages only, mainly English. Whether this hypothesis generalizes to wider populations using different languages remains unknown. To fill in this gap in knowledge, this study investigates the extent to which Generalist Genes could account for individual variation in learning Chinese, which has very different characteristics from English, being a tonal language with a logographic script. Having lexical tones is one of the major unique characteristics of Chinese compared to English. A Chinese syllable in different lexical tones represents different meanings and different characters. Also, the print–sound mappings are ambiguous in Chinese. These unique characteristics make research on Chinese prominent, because many of the research findings on English may not apply in Chinese, and the comparisons of research findings on English and Chinese are ideal in indicating universal or language-specific findings and theories.

In English, the development of language and reading skills is interwoven, and these skills share genetic origins as shown in twin research (e.g. Thompson, Detterman & Plomin, 1991). The genetic overlap between language and reading abilities is substantial, with genetic correlations ranging from .63 to 1.00 (Davis, Haworth & Plomin, 2009; Gayán & Olson, 2003; Haworth, Kovas, Harlaar, Hayiou-Thomas, Petrill, Dale & Plomin, 2009; Hohnen & Stevenson, 1999; Thompson *et al*., 1991). In other words, 63%–100% of the genetic factors influencing language abilities contributed to reading skills. These genetic links are also evidenced in extreme groups (e.g. Hayiou-Thomas, Harlaar, Dale & Plomin, 2010), with genetic correlations varying from .44 to .64 in a lower performance group and .52 in a higher performance group (Bishop, 2001; Haworth, Kovas *et al*., 2009; Haworth, Dale & Plomin, 2009). It is noteworthy that these studies tapped language and reading using different assessments that tested different aspects of ability, but still moderate to strong genetic overlap was consistently demonstrated. Moreover, these genetic links contributed not only to concurrent relationships but also longitudinal links between language and reading abilities. Specifically, early language abilities at 2 to 4.5 years of age shared sources of genetic influence with subsequent reading skills at 7 to 10 years of age (Harlaar, Hayiou-Thomas, Dale & Plomin, 2008; Hayiou-Thomas *et al*., 2010; Hayiou-Thomas, Harlaar, Dale & Plomin, 2006).

The effects of Generalist Genes extend from within verbal skills to across verbal and nonverbal abilities. Research has demonstrated that general cognitive abilities had strong genetic correlations with language skills (over .63) and reading skills (over .61) in unselected samples (e.g. Colledge, Bishop, Koeppen-Schomerus, Price, Happé, Eley, Dale & Plomin, 2002; Davis *et al*., 2009; Haworth, Kovas *et al*., 2009; Thompson *et al*., 1991). General cognitive abilities shared genetic origins with language-related cognitive skills, such as phonological awareness (Hohnen & Stevenson, 1999; Gayán & Olson, 2003). In sum, converging research evidence has shown that nonverbal cognitive, language and reading skills share genetic underpinnings, which supports the Generalist Genes Hypothesis.

### Research questions

This study investigates the etiology of general cognitive, language and reading abilities and their overlap in 312 Chinese twin pairs aged from 3 to 11. The analysis reported here used the same sample as (Chow, Ho, Wong, Waye & Bishop, 2011), who reported results of univariate genetic contributions to various language and reading measures. Here we addressed a further question. Do nonverbal cognitive, language and reading abilities share common sources of genetic influence, supporting the notion of Generalist Genes? If yes, do Generalist Genes apply across nonverbal cognitive, language and reading skills? Or are common genetic underpinnings found for verbal skills only?

## Method

### Participants

The sample included 339 pairs of typically developing Chinese twins aged from 3 to 11. The mean age was 6.71 years, and the age distribution is shown in Table 1. All participants were kindergarteners and primary school students. In Hong Kong, the majority of children (98.4%) start learning to read Chinese characters at age 4 or below (Li & Rao, 2005). These Cantonese-speaking twins were of the same sex. SNP testing was conducted to determine twin pairs' zygosity, and an audiometric screening test was administered to test hearing ability of speech frequencies. The final sample included 312 twin pairs who could hear 35 dB or above with the better ear. There were 228 pairs of monozygotic twins (116 male pairs and 112 female pairs) and 84 pairs of same-sex dizygotic twins (50 male pairs and 34 female pairs). The same-sex dizygotic to monozygotic twin ratio was 0.37. In fact, the DZ and MZ twinning ratio tends to be lower in Asian populations (Imaizumi, 2003). The proportion of twin types in our study was comparable to that of the population prevalence as found in past studies (Chia, Lee, Cheung, Cheung, Seielstad, Wilcox & Liu, 2004).

**Table 1:** Age distribution of the participants

Age	Frequency (Twin pairs) (Percentage in parentheses)
3–4	17 (5.45%)
4–5	57 (18.27%)
5–6	58 (18.59%)
6–7	43 (13.78%)
7–8	47 (15.06%)
8–9	49 (15.71%)
9–10	21 (6.73%)
10–11	20 (6.41%)
Total	312 (100%)

### Measures

All measures were constructed to be suitable for children across this age range. Pilot testing was conducted on 90 children of this age range to ensure that the measures were suitable for tapping the variability. Items were ranked in increasing difficulty. Also, a ceiling rule was set for most of the tasks.

#### Word reading

The word reading test consisted of a 48-item character reading list and 150 items adapted from the reading subtest of the Hong Kong Test of Specific Learning Difficulties in Reading and Writing (HKT-SpLD; Ho, Chan, Tsang & Lee, 2000). The child was required to read each word aloud. These items were ranked in increasing difficulty, and testing stopped when the child failed 15 consecutive items. The maximum score was 198, and Cronbach's α was .996.

#### Receptive vocabulary

The receptive vocabulary test consisted of 80 test items translated and adapted for Chinese from the Peabody Picture Vocabulary Test - Fourth Edition (PPVT-IV; Dunn & Dunn, 2007). For each item, the experimenter read out the target item and the child was required to select a picture from the four options to match it. These items were ranked in increasing difficulty. Correct responses given on nine or all items of the first 10 consecutive items fulfilled the basal rule. Testing stopped when the child failed to read 15 consecutive items. The maximum score was 80, and Cronbach's α was .96.

#### Phonological memory

A nonword repetition task consisted of nonword strings ranging from two syllables to seven syllables. A nonword string was constituted by combinations of Cantonese syllables which had no lexical meaning as a whole (e.g. 加坤 */ga1 kwan1/*). For each of the 14 items, the child was presented with a nonword string, and was then requested to repeat the nonword string. For each test item, a point was given for each correct syllable and also for each correct order of consecutive syllable pairs, but a point was deducted for each excessive syllable. Testing stopped when the child failed four consecutive items. The maximum score was 124, and Cronbach's α was .90.

#### Tone awareness

The computer-administered Cantonese tone task consisted of three blocks of items arranged in the following order: three-syllable, two-syllable, and one-syllable blocks. For each of the 15 items, the child was presented with three pictures each illustrating a syllable which had a different tone from the others. The child was required to label each of them, and was given the syllables if they were not able to label them correctly. The child was then presented with a meaningless Cantonese tone sound, and was then asked to select the syllable which matched with the sound. For instance, a meaningless Cantonese second tone sound was presented and the three options were 褲 (*/fu3/* trousers) 碗 (*/wun2/* bowl), and 船 (*/syun4/* ship). The answer was 碗 (*/wun2/* bowl) which has a second tone. The maximum score was 15, and Cronbach's α was .66.

#### Syllable and rhyme awareness

This test was composed of the syllable deletion and rime detection tasks. *The syllable deletion task* consisted of three blocks of items in an increasing difficulty order: real words, nonwords (i.e. syllables which had no lexical meaning as a whole; e.g. 美小 */mei5 siu2/* in which 美 means beauty and 小 means small, but 美小 had no lexical meaning as a whole) and nonsense words (i.e. nonsense syllables which had no lexical meaning individually or as a whole; e.g. */wei1 nu6/* in which */wei1/, /nu6/,* and */wei1 nu6/* have no lexical meaning). For each of the 15 items, the child was orally presented the word/nonword/nonsense word, and was asked to delete a syllable from it. For example, 美小 */mei5 siu2/* without ‘美’ /*mei5*/ is ‘小’ /*siu2*/. *The rime detection task* consisted of nine test items. For each item, the experimenter read out a target syllable, and then read out three syllables each of which was illustrated by a picture. The child was required to select a syllable from the three options which rhymed with the target syllable. The maximum score of the combined task was 24, and Cronbach's α was .88.

#### Rapid automatized naming

The rapid automatized naming task consisted of 6 rows of 5 digits (2, 4, 5, 7, and 9). These digits were arranged in a different order for each row. The child was asked to name all digits at the fastest speed possible. Two trials were completed, and the average time was recorded. The measure for analyses was (1/ average time). Therefore, a higher score indicated better rapid automatized naming, proportional to the number of words read per unit time.

#### Morphological awareness

This test consisted of three tasks of morphological awareness arranged in order of increasing difficulty. Testing stopped when the child failed four out of five consecutive items. *The receptive morphological awareness task* had 10 items. For each item, a novel concept created by a combination of morphemes was orally presented to the child. The child was then required to select a picture from the five options which illustrated the target item. For example, the novel concept was a striped elephant (斑象 /*baan1 zoeng6*/) and the five picture options were (a) a zebra (斑馬/*baan1 maa5*/), (b) a striped dog (斑狗 /*baan1 gau2*/), (c) stripes and an elephant (斑+象/*baan1*/ + /*zoeng6*/), (d) a dog and an elephant (狗+象/ *gau2*/ + /*zoeng6*/), and (e) a striped elephant (斑象 /*baan1 zoeng6*/). *The morphological construction task* had 12 items. For each item, a scenario was orally presented by the experimenter, and the child was asked to construct words for the novel objects or concepts according to the scenarios. For instance, ‘An island that is full of yellow chrysanthemums, is called a yellow chrysanthemum island (黃菊島 /*wong4 guk1 dou2*/). What shall we call an island that is full of red peach blossom?' The target answer was a red peach blossom island (紅桃島 /*hung4 tou4 dou2*/). A target answer was awarded two points, and a partially correct answer was awarded one point. *The homophone task* consisted of five items. For each item, a character was orally presented in the context of a word, and the child was asked to produce words including this character in 10 seconds, and then produce words including the homophones of this character in 10 seconds. For example, the target character is ‘兒’ of the word ‘兒童' (children /*ji4 tung4*/). The words constituting this character can be ‘兒子' (son /*ji4 zi2*/), while the words composed of its homophones can be ‘懷疑’ (suspect /*waa14 ji4*/). The score of the combined tasks was 44, and Cronbach's α was .90.

#### Orthographic skills

Two tasks of orthographic skill adapted from the Hong Kong Test of Specific Learning Difficulties in Reading and Writing (HKT-SpLD; Ho *et al*., 2000) were used. *The left–right reversal task* consisted of 21 simple Chinese characters and 4 alphabetic numbers, of which 14 of them were left–right reversed (e.g. the character 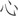 was left–right reversed as 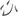). The lexical decision task consisted of 30 rare real characters, and 30 noncharacters with radicals placed in illegal positions. The child was required to cross out the items with an incorrect orientation or the noncharacters. A point was given for an item identified correctly, i.e. an incorrect oriented item/ noncharacter was crossed out or a correct oriented item/ real character was left uncrossed. The maximum score of the combined task was 85, and Cronbach's α was .93.

#### Nonverbal cognitive ability

The Raven's Colored Progressive Matrices (RCPM; Raven, Court & Raven, 1995) was employed to assess nonverbal reasoning. The RCPM consisted of three sets of 12 items. All 36 items were used in this study. For each item, the child was required to choose from six options the best one to fill in the missing part of a matrix. As this task had not yet been normed in the Chinese population, raw scores were used. The two trial items were not included in the total score. The maximum score was 34, and Cronbach's α was .93.

#### Procedure

Each child was tested in a 1-hour session by trained psychology major undergraduates and graduates in their school, their home, or our research laboratory in Hong Kong according to parents' preference. The majority of participants were tested at their school or home. Saliva was collected from co-twins with DNA kits for zygosity assessment.

## Results

To adjust for age effects, the raw scores of each task were regressed on children's age, and the standardized residuals represented children's ability in each domain. These standardized scores adjusted for age were employed in analyses. To test the size of sex differences on the measures, *R*^*2*^ between sex and age-adjusted scores were computed, and they ranged from .00 to .01. This indicates that sex effects were not large enough to influence intraclass correlations and therefore no statistical correction for sex effects was applied (McGue & Bouchard, 1984).

### Factor structures of language and reading measures

An exploratory factor analysis was employed on the scores of one twin randomly selected from each twin pair to explore the dimensionality of the eight language and reading tasks using SPSS. The factor solution was then tested with a confirmatory factor analysis on the scores of the other twin in each twin pair using Amos. The aim of these factor analyses was to indicate the factor structures of the language and reading skills. Nonverbal cognitive ability was kept separate because nonverbal ability is typically regarded as distinct from language and reading, and the genetic overlap between nonverbal and verbal skills could be examined with greater precision in genetic analyses. The exploratory factor analysis indicated two factors with eigenvalues of more than 1. Direct Oblimin rotation showed items that clustered on two factors (see Table 2). Receptive vocabulary, phonological memory, tone awareness, syllable and rhyme awareness, and morphological awareness had moderate to high loadings on the first factor, which was referred as Language. Word reading, rapid automatized naming and orthographic skills were moderately to highly loaded on the other factor, which we termed Reading. The confirmatory factor analysis showed that this two-factor solution had a significant chi-square (*χ*^*2*^ = 52.86, *df* = 19). However, the chi-square index is sample-size sensitive, and thus for a sample size of over 300 data, a significant chi-square may be due to the large sample size rather than a poor fit model. So, other model fit indexes are more relevant. The two-factor solution had an adequate goodness of fit (SRMR = .046; NFI = .92; RMSEA = .08; Hoslter = 181). The factor scores were the summed *z*-scores of the individual tests loading on each factor.

**Table 2:** Rotated factor loadings of exploratory factor analysis with direct oblimin rotation on scores controlling for age

Variables	Language	Reading
Receptive vocabulary	**.71**	.06
Phonological memory	**.67**	−.10
Tone awareness	**.63**	−.11
Syllable and rhyme awareness	**.61**	.28
Morphological awareness	**.72**	.17
Word reading	.07	**.72**
Rapid automatized naming	−.15	**.80**
Orthographic skills	.17	**.63**

*Note*. Factor loadings over .4 are bolded. N = 312.

### Genetic analyses

Results of independent sample *t*-tests on the scores of one twin randomly selected from each twin pair showed that MZ twins and DZ twins did not differ significantly on the two factors. The scores were first fitted to the univariate ACE models and the Cholesky decomposition models by OpenMx in the R statistical modeling package (Boker, Neale, Maes, Wilde, Spiegel, Brick, Spies, Estabrook, Kenny, Bates, Mehta & Fox, 2011).

#### Univariate genetic analyses

**Figure 1:**
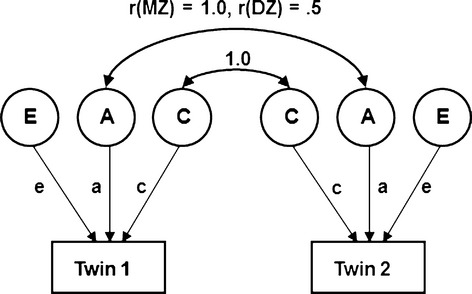
Univariate ACE model.

A univariate ACE model estimates the proportion of phenotypic variance due to genetic (A), shared environmental (C) and non-shared environmental (E) factors (see Figure 1). The two models provided satisfactory goodness-of-fit (*p*s > .05). Substantial genetic influences were found for Nonverbal Cognitive Ability, explaining 70% of its variance. Also, moderate genetic influences were shown for Language and Reading, explaining 32% and 49% of their variance, respectively. The correlations of co-twins' scores by zygosity and the parameter estimates are shown in Table 3.

**Table 3:** Twin correlations by zygosity and genetic model parameter estimates of measures controlling for age (95% confidence Intervals in parentheses)

Variable	Twin correlations		ACE models				
	
	MZ	DZ	a^2^	c^2^	e^2^	*∆χ*^*2*^ (*∆df*=6)	*p*
Nonverbal Cognitive Ability	.71	.37	.70 (.61, .80)	.00 (.00, .00)	.30 (.26, .33)	5.84	.44
Language	.80	.65	.32 (.15, .48)	.48 (.31, .66)	.20 (.17, .23)	6.15	.41
Reading	.74	.50	.49 (.27, .72)	.25 (.03, .47)	.26 (.22, .29)	1.53	.96

*Note*. MZ = 210 to 227 pairs; DZ = 80 to 84 pairs. a^2^ = additive genetic variance; c^2^ = shared environment variance; e^2^ = nonshared environment variance; *∆χ*^*2*^ and *∆df* are the differences between the saturated and the ACE models.

#### Multivariate genetic analyses

**Figure 2:**
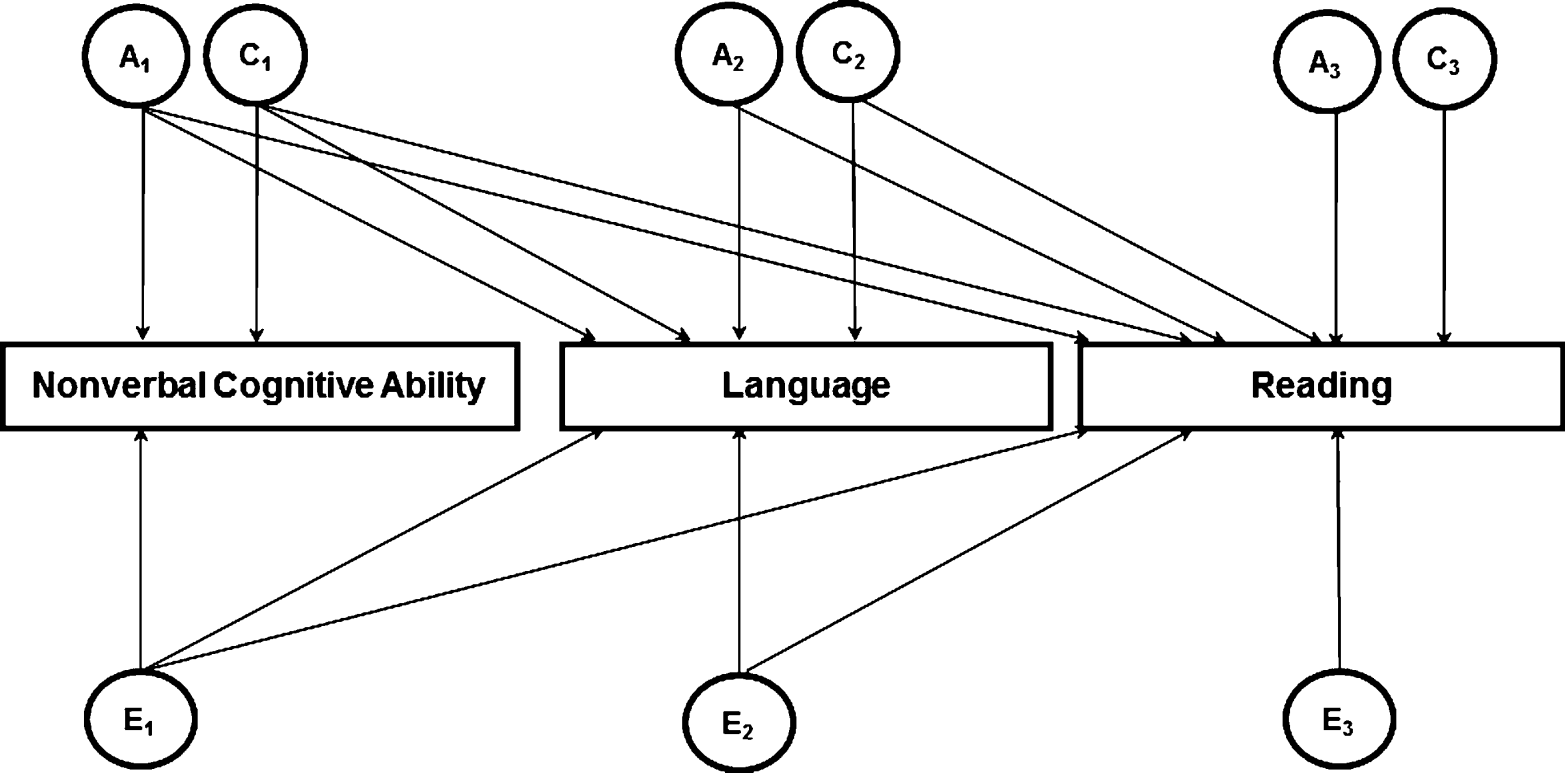
Cholesky decomposition model.

The Cholesky decomposition model was employed to investigate the genetic and environmental links between Nonverbal Cognitive Ability, Language and Reading (see Figure 2). Three sets of ACE terms were specified in the model. The first set (A1, C1 and E1) linked to Nonverbal Cognitive Ability, Language, and Reading, and the second set (A2, C2, and E2) linked to Language and Reading. The third set (A3, C3, E3) contributed to Reading only. This model yielded a good fit (model fitting statistics of this model compared with the saturated model, ∆−*2LL*[∆*df* = 33] = 29.31, *p* > .05).

Results indicated significant paths linking A1 to Nonverbal Cognitive Ability, Language and Reading, indicating their common genetic origins (see Table 4). However, after the genetic influences shared with Nonverbal Cognitive Ability were accounted for, there were no further common genetic influences between Language and Reading (i.e. nonsignificant path linking A2 to Reading). The genetic correlations varied from .47 to .58 (.58 for Nonverbal Cognitive Ability and Language; .47 for Nonverbal Cognitive Ability and Reading; and .48 for Language and Reading). Hence, 47% to 58% of the genetic influences overlapped across the skills.

**Table 4:** Standardized path coefficients from a Cholesky decomposition model of genetic (A); shared-environment (C); and nonshared-environment (E) influences on general cognitive ability, language and reading controlling for age (95% confidence intervals in parentheses)

Variables	Paths		

	A1	A2	A3
Nonverbal Cognitive Ability	0.76 (0.64,0.88)		
Language	0.44 (0.16,0.73)	0.43 (0.11,0.75)	
Reading	0.36 (0.20,0.52)	0.14 (−0.15,0.42)	0.45 (0.24,0.66)
	C1	C2	C3
Nonverbal Cognitive Ability	0.34 (0.11,0.58)		
Language	0.95 (0.70,1.19)	0.26 (−0.40, 0.92)	
Reading	0.03 (−0.42,0.47)	0.39 (0.15,0.63)	0.00 (−9.57,9.57)
	E1	E2	E3
Nonverbal Cognitive Ability	0.55 (0.50,0.60)		
Language	0.06 (−0.02,0.14)	0.03 (−0.07,0.12)	
Reading	0.35 (0.32,0.38)	−0.06 (−0.11,0.00)	0.39 (0.36,0.43)

The shared environmental link between Nonverbal Cognitive Ability and Language, and nonshared environmental link between Nonverbal Cognitive Ability and Reading, were significant. However, other environmental links were not significant, including those between Language and Reading, indicating their unique environmental origins.

## Discussion

This study investigated the common etiology of nonverbal cognitive, language and reading abilities in 312 Chinese twin pairs aged from 3 to 11. There are three major findings. First, various measures of language and reading abilities yielded two factors. Specifically, receptive vocabulary, phonological memory, tone awareness, syllable and rhyme awareness, and morphological awareness formed a factor, which was referred as Language. Word reading, rapid automatized naming and orthographic skills formed another other factor, which represented Reading. Second, results that showed genetic influences were substantial for Nonverbal Cognitive Ability, and moderate for Language and Reading. Lastly, Nonverbal Cognitive, Language, and Reading abilities shared genetic origins, supporting the Generalist Genes Hypothesis. Therefore, the general effects of genes on various cognitive skills could extend to Chinese which has very different characteristics from alphabetic languages, suggesting the universality of the Generalist Genes Hypothesis. However, Language skills did not share environmental origins with Reading abilities. These findings have clarified the etiology of the links between language and reading skills in Chinese.

### Genetic factors contributed to individual differences in cognitive skills

Significant genetic influences were demonstrated across measures. Specifically, substantial genetic influences were found for Nonverbal Cognitive Ability, which explained 70% of its variance. Past studies found moderate to strong genetic influences for nonverbal cognitive ability in English-speaking children, though relatively smaller estimates were shown in younger children (e.g. Colledge *et al*., 2002; Thompson *et al*., 1991). Also, moderate genetic influences were found for Language and Reading abilities, explaining 32% and 49% of their variance, respectively. Past studies have indicated moderate genetic effects on language factors, and moderate to strong genetic influences on reading factors, even though these factors represented various facets of language- or reading-related skills (e.g. Colledge *et al*., 2002; Harlaar *et al*., 2008; Hohnen & Stevenson, 1999). So, genetic factors play a role in language and reading skills across languages.

### Genetic overlap but environmental independence

Multivariate genetic analyses showed a significant genetic link between Nonverbal Cognitive Ability and Language and Reading skills. Moderate genetic correlations were found (genetic correlations ranged from .47 to .58). These findings indicate that as well as genetic influences specific to each domain, there are general effects of genes on cognitive skills that extend beyond alphabetic languages. Measures that are important to one language might be less relevant in another. For instance, tone awareness was tested in our study as Chinese is a tonal language, while it is not included in studies on English skills. Despite the fact that different facets of language skills were included, common genetic origins were indicated, underscoring the universality of Generalist Genes. Also, these findings are similar to past research studies with samples of a different age range and language ability. For instance, Hayiou-Thomas, Kovas, Harlaar, Plomin, Bishop and Dale (2006) found both common and specific genetic effects across the two factors representing various verbal measures, namely language and articulation, in 4.5-year-old twins with language difficulties. These imply that Generalist Genes may apply in different populations. The findings of common genetic underpinnings raise questions about underlying mechanisms. One possibility is that there are genes that have a targeted effect on development of focal brain regions, such as prefrontal cortex, which are important for a wide range of verbal and nonverbal cognitive skills. Another possibility is that general effects reflect the influence of genes that influence developmental processes such as neuronal migration, synaptic pruning or myelination across a wide range of cortical regions.

The nonsignificant environmental links between Chinese Language and Reading skills indicate that different kinds of environmental influences are important for oral and written language. This echoes with past research findings on the specific impacts of home and classroom literacy experiences on either language or literacy skills in Chinese. For instance, a recent training study found that shared book reading enhanced Chinese language skills, whereas parents' explicit metalinguistic training better prepared children for learning to read Chinese (Chow, McBride-Chang, Cheung & Chow, 2008). Also, only formal literacy experiences, such as instruction in reading and writing, were linked to Chinese literacy abilities, but informal literacy experiences, such as storybook reading, did not (Li, Corrie & Wong, 2008). These research findings also chime with studies on children learning English as a mother tongue (e.g. Sénéchal, 2006), and support the Home Literacy Model (Sénéchal & LeFevre, 2002), which proposed various home literacy experiences linked to language and literacy abilities differently. They inform parents and educators that various activities may be optimal for language-related or print-related skills, respectively. The extent of environmental variability in the sample will also affect the likelihood of observing significant environmental links. Further research can incorporate direct measures of some of the environmental factors discussed above to assess the amount of home and school variation in a sample and its influence on twin similarity.

The nonshared environment term of the ACE model incorporates effects of long-term twin-specific influences as well as transient or random factors affecting test performance (measurement error). Overall, these effects, like those of shared environment, were separate for Language and Reading factors.

In sum, our results have provided evidence for the general effects of genes on various cognitive skills and special influences of environment on language and reading abilities. We kept nonverbal cognitive ability separate because nonverbal ability is typically regarded as distinct from language. It is noteworthy that, in this dataset, language and nonverbal cognitive abilities clustered together to the extent that if nonverbal cognitive was included in the initial factor analysis, just two factors emerged, one with loadings from the language measures plus nonverbal reasoning, and the other with reading measures. In fact, different cognitive measures forming one general ability factor gives further support to the Generalist Genes account.

### Limitations

Findings of this study should be interpreted in the context of three caveats. First, the present study included children of a relatively wide age range. Therefore, it should be noted that this study indicated the general patterns of children's individual variations in Chinese language and reading skills, instead of those in a particular developmental stage. It is noteworthy that converging research evidence suggests that heritability of cognitive ability increases across age (Haworth, Wright, Luciano, Martin, de Geus, van Beijsterveldt *et al*., 2010). A much larger sample would be needed to investigate the heritability change or stability in this age range. Second, genetic effects on specific language and reading skills were not tested in this study. Very large samples would be needed for an adequately powered multivariate analysis with many variables and so we focused instead on core constructs of language and reading based on a range of measures. Third, our sample size is not large enough to detect small environmental effects. For instance, our sample size gave power of .73 to detect c^2^ of .4 in a univariate analysis when a^2^ is fixed at .4.

### Implications and conclusions

This study has enhanced our understanding of the extent to which genetic factors influence various domains of cognitive skills. Specifically, its contributions are threefold. First, this study has demonstrated that genetic factors played a role in various domains of cognitive skills, including nonverbal cognitive ability, and language and reading skills in Chinese children. Second, this study has substantiated the Generalist Genes Hypothesis and has extended it to Chinese language and reading skills. These results suggest that the general effects of genes could be universal across languages, and the possible mechanisms of the comorbidity of difficulties in nonverbal cognitive, language and reading skills. Third, these are helpful for future molecular genetics studies, as genes influencing one domain of cognitive skills are likely to exert effects on another. These findings motivate the construction of efficient learning environments, and suggest that different activities tailor-made to Chinese language or reading learning could be useful.
